
JOURNAL INFORMATION
==============================
NLM Title Abbreviation: Innov Aging
Journal ID: Innov Aging
NLM Journal ID: 101703706
Journal ID: innovateage

Innovation in Aging

EISSN: 2399-5300
Publisher: Oxford University Press

ARTICLE INFORMATION
==============================
PMCID: PMC6844781
DOI: 10.1093/geroni/igz038.2823
Article ID: igz038.2823
Article version: 1
Subjects: Session 3540 (Symposium)

IMMUNITY AND AGING—THE HUMAN FACE

Benayoun Bérénice A Chair
University of Southern California, Leonard Davis School of Gerontology , Los Angeles, California, United States
Publication date: 2019 Nov
Electronic publication date: 2019 Nov 8
Volume: 3
Issue: Suppl 1
Issue title: Program Abstracts from the GSA 2019 Annual Scientific Meeting “Strength in Age—Harnessing the Power of Networks”
First page: S768
Last page: S768
Copyright: © The Author(s) 2019. Published by Oxford University Press on behalf of The Gerontological Society of America.
Copyright year: 2019
License: This is an Open Access article distributed under the terms of the Creative Commons Attribution License (http://creativecommons.org/licenses/by/4.0/), which permits unrestricted reuse, distribution, and reproduction in any medium, provided the original work is properly cited.
License URL: https://creativecommons.org/licenses/by/4.0/

Page count: 1

==============================
### IMMUNITY AND AGING—THE HUMAN FACE

Chair: Bérénice A. Benayoun, *University of Southern California, Leonard Davis School of Gerontology, Los Angeles, California, United States*
